# Allogeneic Stem Cell Transplantation for Acute Myeloid Leukemia: Who, When, and How?

**DOI:** 10.3389/fimmu.2021.659595

**Published:** 2021-05-03

**Authors:** Justin Loke, Richard Buka, Charles Craddock

**Affiliations:** ^1^ Centre for Clinical Haematology, Queen Elizabeth Hospital, Birmingham, United Kingdom; ^2^ CRUK Clinical Trials Unit, University of Birmingham, Birmingham, United Kingdom

**Keywords:** acute myeloid leukemia, allogeneic stem cell transplantation, graft-versus-leukemia, chemotherapy, minimal residual disease, measurable residual disease (MRD)

## Abstract

Although the majority of patients with acute myeloid leukemia (AML) treated with intensive chemotherapy achieve a complete remission (CR), many are destined to relapse if treated with intensive chemotherapy alone. Allogeneic stem cell transplant (allo-SCT) represents a pivotally important treatment strategy in fit adults with AML because of its augmented anti-leukemic activity consequent upon dose intensification and the genesis of a potent graft-versus-leukemia effect. Increased donor availability coupled with the advent of reduced intensity conditioning (RIC) regimens has dramatically increased transplant access and consequently allo-SCT is now a key component of the treatment algorithm in both patients with AML in first CR (CR1) and advanced disease. Although transplant related mortality has fallen steadily over recent decades there has been no real progress in reducing the risk of disease relapse which remains the major cause of transplant failure and represents a major area of unmet need. A number of therapeutic approaches with the potential to reduce disease relapse, including advances in induction chemotherapy, the development of novel conditioning regimens and the emergence of the concept of post-transplant maintenance, are currently under development. Furthermore, the use of genetics and measurable residual disease technology in disease assessment has improved the identification of patients who are likely to benefit from an allo-SCT which now represents an increasingly personalized therapy. Future progress in optimizing transplant outcome will be dependent on the successful delivery by the international transplant community of randomized prospective clinical trials which permit examination of current and future transplant therapies with the same degree of rigor as is routinely adopted for non-transplant therapies.

## Introduction

It is more than sixty years since allogeneic stem cell transplantation (allo-SCT) was pioneered as a novel and potentially curative therapeutic modality in patients with chemotherapy-resistant acute myeloid leukemia (AML) (1, 2). Subsequent analyses have confirmed the role of allo-SCT as the optimal treatment strategy in adults with AML in first complete remission (CR1) consequent upon its ability to reduce the risk of disease relapse by more than 60% compared with intensive chemotherapy alone. Remarkably the magnitude of the augmented anti-leukemic activity of allo-SCT, result from both dose intensification and the genesis of a potent graft-versus-leukemia (GVL) effect, is similar in all biological subtypes of AML (3).

The survival benefit of the augmented anti-leukemic activity of allo-SCT is blunted by its attendant transplant related mortality (TRM). It is therefore essential to a) identify patients whose outcome with intensive chemotherapy (IC) is such that the enhanced anti-leukemic activity of allo-SCT is otiose b) identify patients whose outcome with IC is such that deployment of the enhanced anti-leukemic activity of an allograft should be considered and c) define as precisely as possible the patient population in which allo-SCT can be delivered with an acceptable morbidity and mortality. Thus the identification of patients likely to benefit from allo-SCT requires a dynamic assessment which incorporates both the predicted risk of disease relapse if the patient were to receive IC alone coupled with a prediction of the TRM were the patient to proceed to transplant (4). Accurate prediction of these parameters has been refined by progress in both risk stratification utilizing clinical, cytogenetic and molecular genetic data as well as advances in prediction of the risk of allo-SCT (5–9). Increasingly, randomized controlled trials are informing critical questions concerning relapse risk in patients treated with IC alone (10) and informing the personalization of transplant strategies (11–14). Cooperative transplant trials networks such as the US BMT CTN and the UK transplant cooperative IMPACT will play an increasingly important role in optimizing outcomes after allo-SCT in AML (15).

### Who and When Should Patients With AML Be Transplanted?

The focus of therapeutic endeavor in newly diagnosed AML in recent years has primarily been on improving induction chemotherapy (16, 17). However, the increasing availability of allo-SCT coupled with the recognition that a substantial proportion of patients treated with IC alone are destined to relapse has prioritized the development of algorithms designed to identify patients likely to benefit from allo-SCT in CR1. The advent of more accurate risk stratification, utilizing genetic and measurable residual disease (MRD) analysis, coupled with increased sophistication in predicting and reducing TRM has improved decision making concerning the delivery of optimal consolidation therapy in adult AML (18).

The importance of correctly identifying patients in first CR1 who are likely to relapse is predicated by the poor, incomplete rates of remission salvage, such that a significant proportion of patient who relapse do not reach a second CR (CR2) (19). Furthermore, the use of additional intensive chemotherapy and concomitant infections often result in patients with impaired fitness prior to an allo-SCT in CR2. Studies recurrently show that patients with active disease have poorer outcomes as compared to those patients transplanted in CR, thus this should be a critical goal prior to proceeding to transplant (20, 21). Whilst patients transplanted with CR with incomplete count recovery (CRi) have inferior outcomes to patients with AML in CR, this is as a result of increased non-relapse mortality (NRM) but not necessarily relapse risk (22). Other studies have shown the number of courses of consolidation chemotherapy delivered prior to transplant do not improve patient outcome (23).

#### Who Should Be Transplanted With Refractory or Relapsed Disease?

The aim of therapy in fit adults with relapsed with AML is to proceed to allo-SCT once a 2^nd^ CR has been achieved (24). This is based on studies demonstrating very poor outcomes in patients who are not allografted in CR2 (19, 25, 26). However, there may be a subset of patients with core-binding factor translocated AML who may achieve long term remission with autologous transplantation, or in a minority, salvage chemotherapy (19, 27). A number of prognostic systems exist for patients with relapsed/refractory AML (28, 29) which may help to identify subgroups of patients with AML who are likely to have long-term survival following an allo-SCT. Important factors identified in these prognostic systems include, duration of CR1, age at relapse and cytogenetic risk at diagnosis.

Retrospective analyses of allo-SCT for AML in CR2 have demonstrated overall survival (OS) of 30-60%, with acceptable rates of TRM despite intensive pre-treatment in this cohort of patients (30–32). Results have also been encouraging in the use of alternative donors in transplantation at CR2 (32). A formal comparison of myeloablative (MAC) versus RIC regimens in this setting is not possible, but registry studies show no significant differences in OS between patients treated with the differing conditioning intensities (32). Despite this, in fit younger patients who might tolerate a MAC regimen, this is probably the preferred treatment strategy to reduce further disease relapse which remains the major risk facing this patient cohort.

A particularly challenging group of patients with AML are those with primary refractory disease, defined as failure to achieve remission following two cycles of induction chemotherapy (33). Numerous studies have shown that patients transplanted with active disease have poorer outcomes than those in remission (20, 31, 34). However, studies have demonstrated approximately 20-30% of patients with primary refractory disease may have long term survival after an allo-SCT (35) and recent work has identified risk factors that may identify patients who are likely to have primary refractory disease at an earlier stage (36). In the evolving landscape of genetic stratification, these scoring systems are likely to be refined, and the long term impact of novel salvage options from targeted therapies remains to be seen (37, 38). One recent study underlined the particularly poor outcome of patients with *TP53* mutant AML, when they were transplanted with active disease (39). A challenge in assessments of such genetic risk factors will be the clonal evolution which occurs in patients with AML following treatment (40).

Finally, for patients who relapse following an allo-SCT, the outcome is very poor (41). However, for some patients, especially ones with a durable remission since transplant, and with disease control at the time of second allo-SCT, this procedure may provide an OS at 2 years of 25% (42). In patients who received an unrelated donor transplant, no advantage for change in donor in this setting could be demonstrated.

#### Who Should Be Transplanted in First Complete Remission?

**Figure 1:**
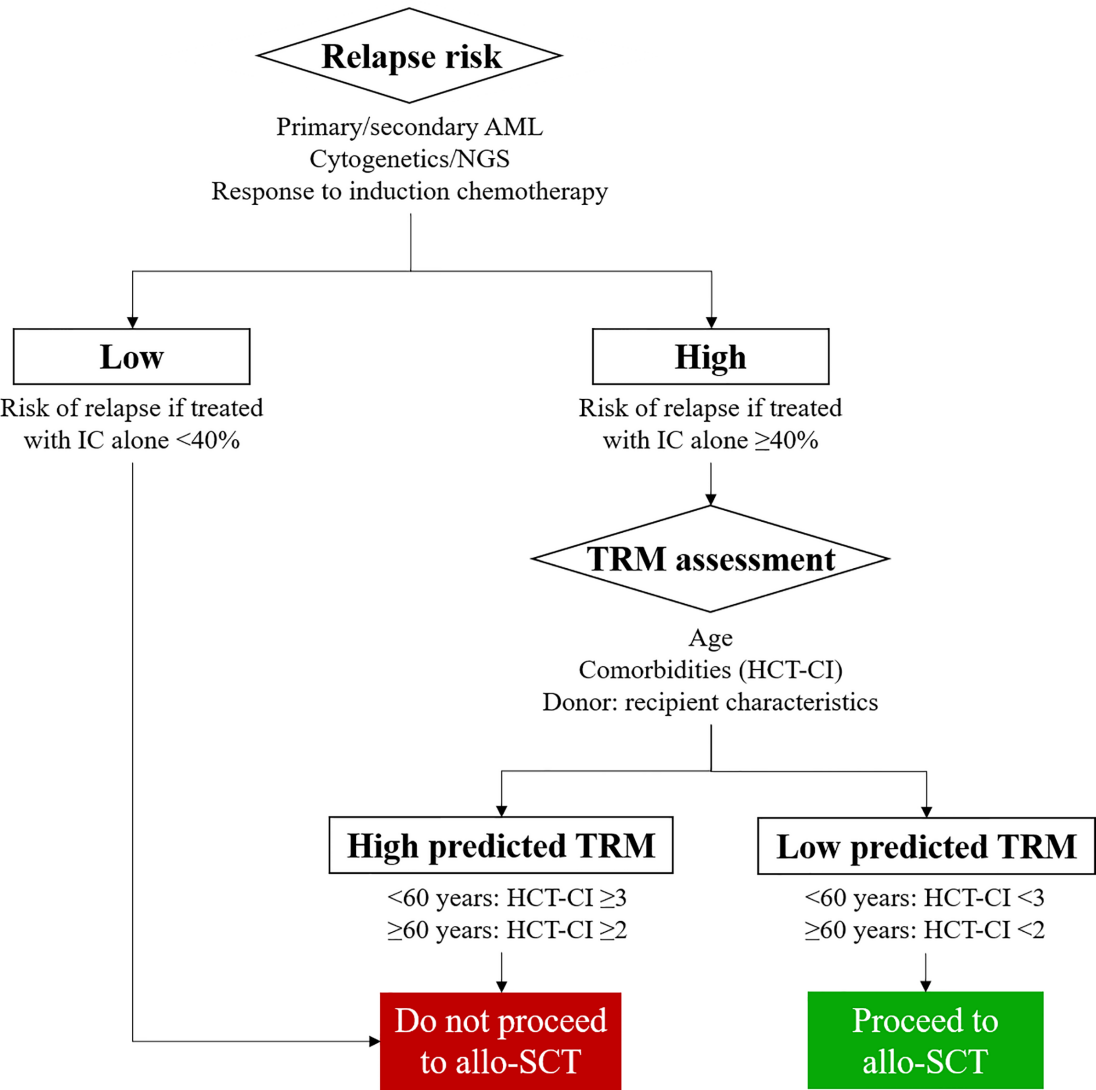
Identifying patients with Acute Myeloid Leukemia (AML) who are likely to benefit from allogeneic stem cell transplantation (allo-SCT). MRD, Measurable Residual Disease; TRM, Transplant related mortality; HCT-CI, Hematopoietic cell transplantation - specific comorbidity index; NGS, next generation sequencing.

Donor versus no donor studies were the first to demonstrate the ability of allo-SCT to increase disease free survival (DFS) and OS in patients transplanted using a myeloablative HLA matched sibling allo-SCT (43). A selection strategy to identify patients who should be transplanted in CR1 was articulated by Cornelissen and colleagues with the European LeukaemiaNet (ELN) AML working party (4) and is based on the competing risks of relapse with chemotherapy alone versus risk of relapse after an allo-SCT and the concomitant TRM (**Figure 1**). Underpinning this treatment algorithm is the observation that the risk of relapse following allo-SCT is more than halved as compared to that observed in patients treated with IC alone (3)- regardless of cytogenetic risk group. At the same time recent reductions in transplant toxicity permit delivery of an allo-SCT with an NRM of 15% or less in fit adults with a well matched sibling or volunteer unrelated donor. On this basis the ELN group recommend consideration of allo-SCT in fit adults with AML in CR1 who have a predicted relapse risk of 35-40% and a suitable donor (33). Thus adults with AML in CR1 who fulfill ELN criteria for good risk disease on the basis of cytogenetics or the presence of an NPM1 mutation without FLT3-ITD mutation, and demonstrate a good response to induction chemotherapy by MRD criteria are not routinely deemed eligible for an allo-SCT in CR1. Conversely, all other adults in CR1 in whom the predicted risk of relapse of >40% if they are treated with IC alone should, in principle, be considered transplant candidates providing a suitable stem cell donor is available (44).

Risk stratification in patients with AML in CR1 is based on clinical (5) factors, such as age and gender, as well as cytogenetic risk based on karyotyping results (6) (**Table 1**). This has been refined in recent years by the discovery of further mutations of prognostic significance in genes such as *FLT3* (45), *NPM1* (46), *ASXL1* (47), *RUNX1* (48) and *TP53* (49) as reflected in the 2017 ELN classification (33). Increasingly mutational information is available for patients as a result of next generation sequencing (NGS) technology assaying panels of commonly affected myeloid genes (33). This is of further importance as these genetic markers are now commonly used as both therapeutic targets (50, 51) and as prognostic markers of response to therapies (52). The results of these large scale sequencing efforts of AML samples at diagnosis, in combination with data relating to treatment use and clinical outcome will likely refine these risk categories. This will provide a “personalized” risk score for individuals patients based on a number of these clinical factors and allow for incorporation of combinations of genetic mutations, such as that seen recently in the study of myeloproliferative neoplasms (53, 54). It is increasingly becoming apparent that both clinical and mutational characteristics determine the kinetics of disease relapse. Importantly patients with a *FLT3* mutation are amongst those likely to relapse early in whom the timing of transplant should not be delayed (55).

**Table 1:** Factors determining disease risk in AML.

Clinical Variables	Molecular variables	Dynamic variables
• Age	• Cytogenetic	• Response to course 1 by morphology
• Gender	• Next generation sequencing of genes e.g. *FLT3, NPM1, RUNX1, ASXL1, TP53*	• Response to treatment by MRD
• Presenting white cell count		
• Primary versus secondary disease		
• Performance status		

##### Incorporation of MRD Risk Stratification

**Figure 2:**
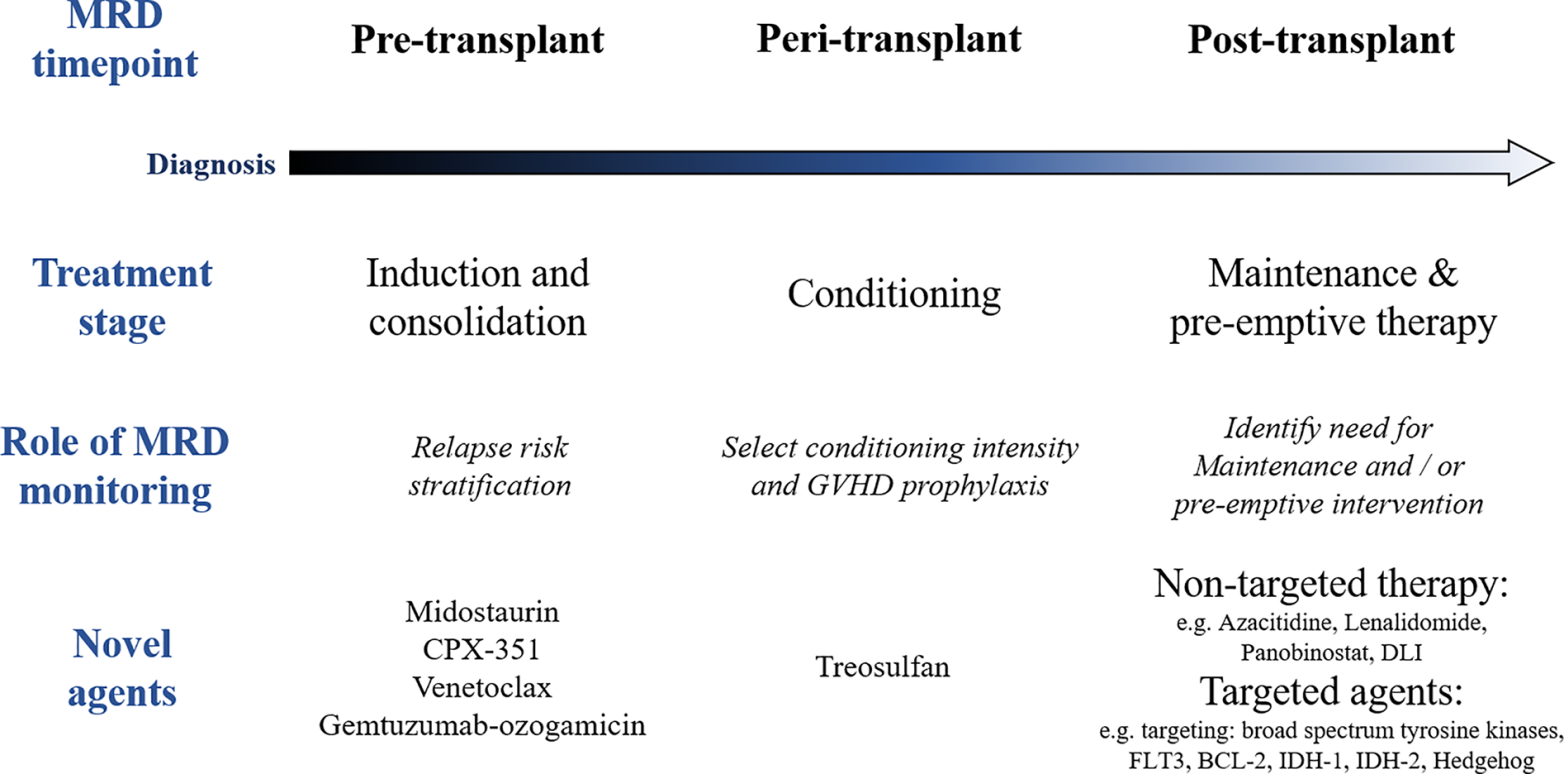
Role of measurable residual disease (MRD) and novel agents at different stages of the treatment pathway in acute myeloid leukemia (AML). GVHD, graft versus host disease; DLI, donor lymphocyte infusion.

An important development in risk stratification has been the incorporation of MRD monitoring to routinely assess patients’ response to chemotherapy (56) (**Table 2** and **Figure 2**). The kinetics and depth of response has been identified as being critical in re-assessing the risk of relapse in patients with otherwise favorable and intermediate risk disease. The impact of MRD monitoring appears to be the most important, independent prognostic factor in many scenarios (57, 58). The selection of the optimal MRD monitoring modality depends on the presence of leukemia specific molecular, cytogenetic or immuno-phenotypic dependent on the AML subtype. Each MRD monitoring technique has its own advantages and disadvantages, and all require expertise in the delivery of reliable results (**Table 2**).

**Table 2:** Relative merits of different MRD monitoring methodologies.

Method	Multi-parameter Flow Cytometry (MFC MRD)	Quantitative PCR (RQ-PCR)	Next generation sequencing (NGS)
**Advantages**	Rapid results Widely applicable to many patents	Sensitive Easily compared with sequential results due to quantitative range Widely accepted standardisation	Applicable to many patients Error correction increases sensitivity
**Disadvantages**	Reliant on expertise of reporting lab Phenotype of AML cells may change over time	Restricted molecular targets (e.g. Core binding factor translocations, NPM1c mutant)	Ongoing development of technology Expense
**Examples of use**	Risk stratification in younger adults, post induction chemotherapy, with NPM1 negative AML.	Risk stratification post chemotherapy to determine relapse risk in NPM1 mutant AML.	Pre-transplant MRD monitoring.

##### Examples of Different Uses of MRD Risk Stratification

Real-time quantitative polymerase chain reaction (RQ-PCR) monitoring of disease specific transcripts provides a sensitive and disease specific assay of MRD for patients with AML expressing a detectable fusion gene transcript (e.g. Core-binding factor (CBF) fusion gene, KMT2A fusion genes, mutant NPM1). In the case of AML with CBF translocation, although age can influence prognosis (59), the depth of response to course 1 and 2 of IC (57) are critical determinants of relapse risk. In cases with residual levels of CBF fusion transcripts at the end of treatment (60), relapse risk depends on level of transcripts, but low levels of CBF fusion gene transcripts may persist after end of treatment without affecting long-term survival. Failure to achieve a 3-log reduction in CBF fusion transcript after two cycles of chemotherapy is associated with an over 50% relapse risk in the monitoring studies of two large cooperative groups, suggesting possible benefit from an allo-SCT in these patients (57, 61).

In younger adults with NPM1 mutant AML, RQ-PCR positivity in the peripheral blood after two cycles of chemotherapy is an important predictor of relapse, identifying a population of patients who should be considered allo-SCT mandatory (58). This is supported by data which points to the beneficial effect of allo-SCT in patients with mutant NPM1 residual disease post induction chemotherapy (62). Recent studies have confirmed that, in younger adults at least, NPM1 is also a predictive biomarker. Patients with NPM1 mutant AML who have a less than 4-log reduction in peripheral blood NPM1 MRD levels demonstrated improved survival after allo-SCT compared with patients who received chemotherapy alone (62). The low relapse risk for patients who are negative for mutant NPM1 transcripts in the peripheral blood after two cycles of intensive chemotherapy outweighs other poor prognostic factors such as concomitant FLT3-ITD mutation or poor risk genotypes (7). The degree to which NPM1 mutations are a prognostic or predictive biomarker in older patients (over the age of 60 years) remains unclear (63). In part this may be due to the increased association of other poor risk cytogenetic features in more elderly patients with NPM1 mutant AML (64). Of note in patients with adverse risk cytogenetics, the presence of NPM1 mutation has no impact on survival outcomes.

A number of large prospective studies have confirmed the prognostic significance of multi-parametric flow cytometry (MFC) determined MRD in adults with newly diagnosed AML treated with IC. In younger adults, MFC MRD+ positive patients with standard risk, NPM1- mutated AML appeared to benefit from allo-SCT in CR1 (10) and data on this group of patients continues to be accrued, including the benefits of intensifying chemotherapy in patients with a suboptimal MRD response after first course of intensive chemotherapy. In older patients, a higher level of MRD after induction treatment is also prognostic of a worse outcome (65). However, in this age group, although MFC MRD negativity, offered improved overall survival, relapse rates remained high.

Early studies suggest a promise for NGS technology for MRD assessment (66), which has the advantage that it may be applicable for many forms of AML. Error correction methodology has become incorporated in this technology to enable higher levels of sensitivity (67), but is currently limited to research settings due to the costs. Furthermore, there has not yet been an upfront comparison of these different MRD technologies independently, or in combination, to compare technical specifications. A recent large study suggested there was an additive prognostic value of NGS MRD over MFC MRD, but interestingly the persistence of age related clonal hematopoiesis after treatment did not result in an increased relapsed rate (66)

##### Improving Assessment of Transplant Related Mortality in Patients With AML

A critical factor to understand whether a patient with AML is suitable for an allo-SCT is estimation of the TRM associated with the procedure and whether it is outweighed by the improvement in relapse risk delivered by the transplant process (68–70) (**Figure 1**). Furthermore, these considerations are central to any discussion with patient and family as to whether the increased risk of an allograft is justifiable. The European Society for Blood and Marrow Transplantation (EBMT) risk score, originally developed in patients allografted for chronic myeloid leukemia (CML) (71), was subsequently shown to be applicable in other disease settings (72), and provided the first attempt to provide a quantifiable estimate of TRM and transplant outcome which could be routinely applied in clinic. However in patients allografted for AML more emphasis is now placed on the Hematopoietic cell transplantation-specific comorbidity index (HCT-CI) score which incorporates a weighted score based on the presence of pre-transplant comorbidities (8). This has been shown to be valid in patients undergoing an allo-SCT for myelodysplastic syndrome (MDS) or AML (9) and more recently combined with age (73), to demonstrate the varying effects of these comorbidities based on a patients’ age. Of note, this analysis showed that younger patients with comorbidities were at a significant disadvantage to older fit individuals with no other significant comorbidities.

Unfortunately no scoring system for TRM can include the importance of a clinical assessment of patients based on the “end of bed” assessment and knowledge of how patients have tolerated recent intensive treatment. Thus despite improvements in mathematical modeling techniques to predict treatment related risk on a personalized basis to account for the dynamic interactions between different variables (74, 75), there remains a considerable limitation in the ability of these scoring systems to predict TRM. Finally, the majority of these scoring systems were developed in the era of sibling or matched unrelated donor transplantation, thereby limiting their use for those with alternative donor sources, which are now of increasing use; such as for recipients of haploidentical donor or umbilical cord stem cells transplants.

##### What Is the Impact of Patients’ Age in Considering Transplant Eligibility?

It is commonly recognized that an important challenge in the management of patients with AML is the increased frequency of this disease with age. Furthermore, the older patient faces a combined challenge of increased frequency of comorbidities and higher risk genetic features (76). Nevertheless patients over the age of 70 years with AML are routinely transplanted with acceptable results (77) but careful assessment of transplant suitability is required. The widely used, updated HCT-CI score allows some adjustments due to age (73), and this analysis showed that younger patients with comorbidities were at a significant disadvantage to older fit individuals with no other significant comorbidities. Nonetheless the HCT-CI score is still of importance in this population, as it has been shown that in patients above 60 years of age a HCT-CI score of 2 or greater results in substantially higher TRM than otherwise expected (78). Future developments to improve assessment of transplant eligibility in this cohort should involve geriatric assessments that encompass an assessment of the functional status of the patient (79).

## How Should Patients With AML in CR1 Be Transplanted?

The major causes of treatment failure in adults allografted for AML are transplant toxicity and disease relapse. Whilst significant progress has been made over recent decades in reducing TRM the risk of disease relapse remains stubbornly high. The key considerations in patients with allo-SCT-mandatory AML include identifying which patients should receive RIC as opposed to a MAC allo-SCT and, in patients lacking a well-matched sibling or unrelated donor, what is the preferential alternative donor stem cell source? The development of strategies with the ability to reduce the risk of disease relapse post-transplant also represents a major unmet need.

### Strategies to Improve Outcomes Pre-Transplant

The design of novel treatment strategies with the potential to reduce the risk of disease relapse post allo-SCT remains a priority if we are to increase the number of patients with AML who benefit from transplant. A number of questions remain regarding the optimal management of patients’ pathway before, during and after an allo-SCT (**Figure 2**). This debate has been reinvigorated in recent years by two key innovations: the widespread use of MRD technologies in patients with AML (80) and the increasing availability of novel pharmacological agents that may be applied at different treatment stages (81) (**Figure 2**). The adverse impact of pre-transplant MRD on post-transplant outcomes has been increasingly widely recognized (14, 82) and this may inform pre-transplant treatment strategies. Furthermore, emerging data suggest that conditioning intensity and potentially graft-versus-host disease prophylaxis strategies may influence the poor prognostic impact of pre-transplant MRD (83). Finally, post-transplant monitoring of MRD may become important in identifying patients who should receive pre-emptive treatment (84) and is likely to be important in future maintenance strategies in patients post allo-SCT.

#### How Important Is Pre-Transplant MRD?

A number of retrospective studies have demonstrated the adverse prognostic significance of patients with MFC MRD positivity prior to transplant (82), with some likening the outcomes of these patients post allo-SCT to those with active disease (85). This draws comparison to the outcomes of younger adults with a partial response to the first cycle of induction chemotherapy who have a similar overall outcome as compared to patients who have a CR or CRi but have MFC MRD positivity (10). Two prospective studies have demonstrated the importance of pre-transplant MRD (14, 83) in patients with AML or high risk MDS. The FIGARO study investigated the impact of pre-transplant MFC MRD in 244 patients entered into a randomized comparison between FLAMSA-Bu-RIC regimen and a control RIC arm. This identified a poor prognostic impact of a 0.2% threshold of residual disease. However, even in the MRD positive arm, only approximately 50% of patients relapsed: not only suggesting further strategies to identify patients at risk of relapse are required (14), but contrary to previously held opinions, this sizeable proportion of patients with high risk AML may be salvageable with an allo-SCT.

The importance of pre-transplant MRD persists regardless of the technique used to monitor MRD. RT-PCR monitoring of CBF fusion transcripts prior to allo-SCT for patients in CR2, show that those with MRD negativity have a reduced risk of relapse as compared to those with MRD positive disease pre-transplant (86).

#### Can We Improve Transplant Outcomes in Patients With Evidence of Pre-Transplant MRD?

It remains unknown whether additional courses of chemotherapy or whether further alterations to transplant management in patients with pre-transplant MRD would be of benefit. However, in recent years a number of provocative results have provided impetus to design clinical trials to tackle the poor prognostic impact of pre-transplant MRD.

#### Pre-Transplant Strategies to Alter Impact of Pre-Transplant MRD?

Studies of novel agents in recent years such as midostaurin and the liposomal cytarabine-daunorubicin preparation CPX-351, suggest that the benefits of these drugs may extend to patients who receive an allo-SCT (16, 50) (**Figure 2**). This provides interesting preliminary data that this may be through improving quality of remissions pre-transplant which may in future studies be measured as pre-transplant MRD. In the case of the FLT3 inhibitor midostaurin which was added to intensive induction and consolidation, the overall survival benefit of the addition of midostaurin appeared to persist in the majority of patients who were allografted in first remission. Notably midostaurin was not administered as post-transplant maintenance in this study. Likewise, CPX-351 demonstrated improved remission rates and OS in patients receiving this drug over standard remission induction therapy in patients with secondary AML. In patients who subsequently received an allo-SCT, those who had received CPX-351 had improved survival as compared to those in the control arm, but the numbers in the study were small, and a smaller proportion were in a remission at time of transplant in the control arm (16). Definitive studies including the incorporation of pre-transplant MRD will be important in validating or refuting the role of pre-transplant therapy in influencing pre-transplant MRD status.

In patients with comorbidities and a high chance of induction related death following intensive chemotherapy, in whom a curative pathway is still intended (87, 88), a less intensive approach may be valid prior to transplant. With the increasing availability of venetoclax based regimens, data will likely emerge as to the transplant outcomes of patients who have a remission following these lower intensity approaches as compared to conventional intensive induction regimens. At present, data on this cohort remains limited, as these regimens have been developed in cohorts of less fit individuals in which the overall transplant rates have been low (89). Certainly, it is well established that patients with AML who have non-proliferative disease, or transformed MDS can have durable remissions with azacitidine alone (90), and patients who proceed to transplant in remission may have long term outcomes which is comparable to those who have remissions from IC (91–93). Although, remission rates for patients receiving non-intensive treatment such as Azacitidine are likely to be inferior as compared to conventional induction chemotherapy alone (94–96), it is unclear whether for patients who do remit, pre-transplant MRD levels are affected by treatment intensity, and whether this has subsequent impact on post-transplant outcomes.

#### Can Changes in Conditioning and GVHD Prophylaxis Alter the Impact of Pre-Transplant MRD?

MRD as measured by error corrected NGS was performed in patients with AML who were enrolled onto the BMT CTN 0901 study which performed a randomized comparison of RIC versus MAC regimens (15). In a comparison of patients who were NGS MRD positive pre-transplant, patients who received a RIC regimen had an inferior outcome to those who were MRD negative at the same timepoint (83). In contrast, in patients transplanted with a MAC regimen, levels of MRD pre-transplant did not appear to affect outcomes post-transplant. This suggested that it was possible to alter transplant conditioning to improve outcomes of patients with MRD pre-transplant, but in practice would be limited to younger patients who would be eligible to receive a MAC regimen regardless (see below).

For those with NPM1 mutant transcripts pre-transplant, the risk of relapse post-transplant is increased. However, this is also dependent on the concomitant FLT3-ITD mutation status (97). The identification of T-cell depletion as an adverse risk factor in the whole cohort, and in those with positive NPM1 MRD pre-transplant, suggest a possible transplant strategy that may improve outcomes for this subset of patients.

### Improving Conditioning Regimens for Patients With AML

Transplant conditioning regimens have evolved since the establishment of allo-SCT as a pivotal tool in reducing relapse risk in patients with AML. MAC regimens established the benefits of an allo-SCT in patients with AML (43, 98) but patients over the age of 40 experienced excess toxicity historically. In the last two decades the increased use of RIC regimens has allowed the routine delivery of an allo-SCT to patients over the age of 70 (77). In recent years the efforts of a number transplant cooperative groups have delivered important randomized controlled trials to optimize transplant conditioning regimens to further inform choice of conditioning regimens (12, 15, 99, 100).

#### What Is the Optimal Conditioning Intensity?

A MAC regimen by definition requires the infusion of donor stem cells to rescue recipients from permanent bone marrow aplasia. The original studies in allo-SCT used conditioning regimens based on radiotherapy (1). This established the basic principles required of any conditioning regimen in acute leukemia, which is to allow durable engraftment of donor hematopoiesis as well as the delivery of an anti-leukemic effect, which is in turn related to the intensity of conditioning (101).

Cyclophosphamide (Cy) based conditioning combined with total body irradiation (TBI) or busulphan are acceptable MAC regimens. The development of intravenous preparations of busulphan has improved the pharmacokinetics of this agent (102) and has practical advantages over TBI based regimens. Measuring busulphan pharmacokinetics may help predict optimal doses in conditioning (103). Cy/TBI regimens are still commonly used and may be better for patients with either central nervous system (CNS) disease or myeloid sarcoma. Nevertheless, a pivotal randomized controlled trial that demonstrated the superior tolerability of a Fludarabine/Busulphan (Flu/Bu4: 12.8 mg/kg over 4 days of IV busulfan) combination over a standard Cyclophosphamide/Busulphan combination, with acceptable tolerability in patients up to the age of 65 (12). This has resulted in the Flu/Bu4 regimen being accepted as a standard of care for fit patients where a MAC regimen is desired.

RIC regimens result in varying duration of cytopenias and are defined as containing less than ≤8 Gy Total Body Irradiation (TBI) or ≤8 mg/kg busulfan (104). The optimal RIC regimen has not been established. A number of RIC regimens have been developed over the last twenty years to enable a tolerable conditioning regimen to be delivered in patients due to either comorbidities or increased age, with varying levels of toxicity and anti-leukemic potency (e.g. Flu/Bu2: 6.4mg/kg, 2 days of IV busulphan) (105), and Flu/melphalan (140 mg/m^2^ of IV melphalan on 1 day) (106). The variability in the effectiveness of these regimens are exemplified by two randomized controlled trials (RCT) of RIC regimens. One study which compared the outcomes of a Flu/2Gy TBI regimen with a Flu/Bu2 regimen demonstrated increased TRM but notable decrease in relapse rates with the Flu/Bu2 regimen (107). In contrast, a recent Flu/Treosulfan study showed superior toxicity incidence to a Flu/Bu2 comparison, but is notable for a TRM in the Flu/Bu2 arm that is far in excess of historical expectations (108).

Given the improved tolerability of novel MAC regimens (12) alongside widespread experience with RIC regimens an important question arose as to whether a MAC or RIC regimen should be selected when either is available in high risk MDS and AML (109, 110). Despite this interest it was surprising that two RCTs comparing RIC and MAC regimens closed early to recruitment but did not demonstrate significant differences in relapse free or overall survival (99, 100, 111). In contrast, a Blood and Marrow Transplant Clinical Trials Network (BMT CTN) study (15) which studied a randomized comparison of RIC versus MAC regimens demonstrated a lower rate of TRM, but higher relapse risk resulting in an inferior relapse free survival (RFS) in patients receiving in the RIC arm as compared to those who received a MAC regimen. However, this study is notable for the higher than expected relapse risk in patients who received a RIC regimen.

The high relapse rates associated with RIC regimens, for patients with high risk AML resulted in the development of the FIGARO study, which compared the outcomes of a standard RIC arm with an augmented RIC schedule with sequential chemotherapy (FLAMSA-Bu) which had shown promising results in early studies in patients with primary refractory disease (112). However, this randomized controlled study demonstrated no improvement in relapse risk from the FLAMSA-Bu regimen as compared to a standard control arm (14).

#### GVHD Prophylaxis Strategies

The introduction of Ciclosporin was critical in establishing the deliverability of allo-SCT in patients with acute leukemia (113, 114) reducing the risk of graft-versus-host disease (GVHD). However, studies that demonstrated an inverse relationship between GVHD and relapse risk form the basis of the evidence underlying the GVL (115, 116). Commensurate with this observation, further studies demonstrated a relationship between ciclosporin exposure and risk of relapse, in the context of T-cell depleted allo-SCT (21, 117). Tacrolimus (FK506) has also been compared with Ciclosporin in a number of randomized trials with varying results (118–120), suggesting a reduction in acute GVHD with the use of Tacrolimus but no significant effect on OS or RFS. Other agents such as Sirolimus (121, 122) and Mycophenolate mofetil (123, 124) have also been used either as an addition or substitute for historical Ciclosporin/Methotrexate combination without a definitive improvement in overall outcomes.

*In vivo* T-cell depletion can be achieved by either Anti-thymocyte globulin (ATG) or Alemtuzumab. Studies demonstrate an improvement in risk of acute GVHD without significant changes in OS (125, 126). However a US retrospective study suggested that ATG compromised relapse risk in patients undergoing a RIC allo-SCT (127) which has led to a discrepancy in the uptake of ATG on the two continents (128). More recent data suggest that variations *in vivo* levels of ATG may result in differences in relapse risk as well as NRM (129). It is also important to note that there appear to be different immunosuppressive properties dependent on the source of ATG, which is critical when different studies are compared (130). The humanized anti-CD52 antibody, Alemtuzumab has also been used extensively as a method of *in vivo* T-cell depletion (131, 132), with control of GVHD particularly notable in the HLA-mismatch setting (133). In more recent years, the use of post-transplant Cyclophosphamide which was pioneered for use in the haploidentical donor allo-SCT setting (134) has been used in the volunteer unrelated donor setting (135) but formal assessment in the clinical trial setting is awaited.

The variation in relapse rate from study to study for these different GVHD prophylaxis studies suggest the need to perform adequately powered studies with suitable endpoints, in order to determine the optimal GVHD prophylaxis strategies in AML.

### How to Improve Outcomes of Patients With AML Post-Transplant

#### Improving Monitoring of Disease Post-Transplant

Whilst the cornerstone of post-transplant care remains careful clinical assessment and review, post-transplant disease monitoring to identify patients at risk of relapse, and timely intervention is becoming more important. This is particularly important with the increased use of RIC allo-SCT which is associated with a higher risk of relapse (15). Furthermore, the use of pre-emptive treatment before fulminant hematological relapse may increase the efficacy of interventions such as donor lymphocyte infusion (DLI) or Azacitidine (136–139).

##### MRD Monitoring Post-Transplant

Prior to hematological relapse, the prognosis of which is usually very poor, early disease re-emergence can be detected by several techniques. The ELN guidelines formally recommend monitoring for MRD post-transplant (33). Similar to pre-transplant, the optimal method for monitoring MRD will be dependent on disease characteristics, and availability of technology, and expertise in the treating center. Post-transplant MRD monitoring has prognostic value. For example, the (8, 21) fusion transcript RUNX1/RUNX1T1 is suitable for MRD monitoring and has been investigated post-transplant (60, 140, 141). Similar to pre-transplant, detectable RUNX1/RUNX1T1 transcripts at 3 months after transplant was a more potent predictor of relapse than presence of c-KIT mutations (141). The most prognostic threshold of MRD may be different after transplant, as compared to that of the pre-transplant setting. For example, one study determined the prognostic impact of NPM1 MRD pre- and post-transplant and found that 1% increase in transcripts pre-transplant and a 10% increase post-transplant were predictive of outcome (142). A combination of multiple methods to detect MRD may be required to provide the most accurate prognostic information. For example combining NGS MRD for NPM1 with multicolor flow cytometry may improve relapse prediction over either modality alone (143).

Discrepancies between the most discriminatory MRD thresholds at different treatment stages illustrate how the pre- and post-transplant bone marrow environment is different; post-transplant, there is a complex immunological milieu of developing tolerance and GVL. As not all patients with MRD relapse, it is postulated that the GVL effect may eradicate residual disease without the need for further intervention. Although it is also logical that early intervention for patients with molecular MRD would be beneficial, there is limited evidence to support this strategy. In a sub-analysis of patients included in the UK AML17 trial, the provision of post-transplant MRD information to clinicians did not affect outcomes – although this was not a randomized comparison, and not a main aim of the study (97).

##### Chimerism

Post-transplant monitoring of host-donor hematopoietic chimerism is a widely used post-transplant monitoring strategy, particularly after RIC allo-SCT. Chimerism can be measured in the whole blood, or specifically in T cells (CD3+ selected) or myeloid cells (CD33+). It is known that patients with mixed chimerism post-RIC allo-SCT do have an increased risk of relapse (144), although it should be noted that chimerism and residual disease are conceptually different. Mixed chimerism does not necessarily mean the presence of residual disease, nor does complete chimerism confirm its absence. In haploidentical allo-SCT disease relapse can occur due to acquired uniparental disomy of chromosome 6p leading to loss of the mismatched HLA-haplotype on leukemia cells and subsequent immune escape (145, 146). In this context, chimerism measurement by disparate methodologies can yield different results: recipient non-HLA marker based chimerism shows an increase during relapse, whilst HLA marker based chimerism remains low in disease relapse driven by a loss of HLA (147). Nevertheless chimerism monitoring, post RIC allo-SCT is an important way of identifying patients at high risk of relapse in whom intervention with pre-emptive DLI may be beneficial. Patients who achieve full donor chimerism (FDC) with DLI have a comparable outcome to those who reach FDC spontaneously (148, 149).

There may be ways to improve the performance of chimerism monitoring, including earlier use post-transplant (150), in CD34+ cells (151–154), and, in combination with monitoring for MRD. Waterhouse et al. compared the utility of chimerism and molecular monitoring including WT1 over-expression. Of 15/70 patients in whom increasing mixed chimerism was detected, all had a positive MRD marker and/or increased WT1 expression. They found that in half, detectable MRD and mixed chimerism occurred at the same time but in the other half, mixed chimerism preceded MRD positivity (155). The FIGARO study demonstrated that the risk of relapse following pre-transplant MRD positivity, is reduced by the achievement of full donor chimerism (14), and is a key finding that should direct future treatment strategies to identify methods of increasing the rate of achieving full donor chimerism.

#### Post-Transplant Maintenance Strategies to Reduce Relapse

**Figure 3:**
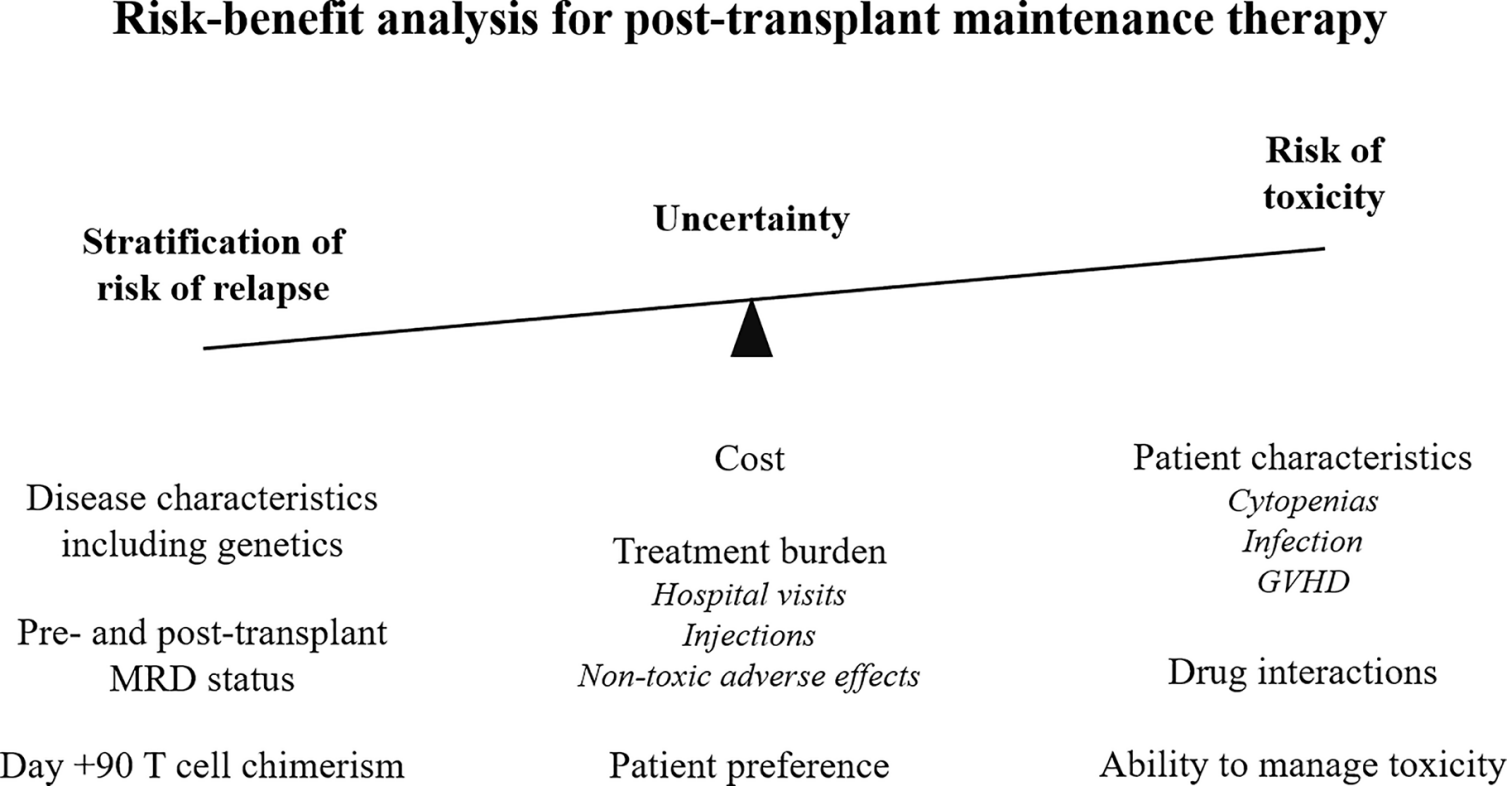
Risk-benefit analysis for maintenance therapy post-allogeneic stem cell transplant. MRD, measurable residual disease; GVHD, graft versus host disease.

Post-transplant pharmacological interventions may have direct activity on malignant cells, and there is improving understanding that modulation of the complex immunological environment may provide additional benefit. There is improving interest in assessing the impact of routine, maintenance treatments, which do not significantly add to the burden of toxicity which includes infection, organ toxicity, and GVHD (**Table 3** and **Figure 3**).

**Table 3:** Examples of post-transplant maintenance strategies.

		Mechanism	Examples of use
**Non-targeted agents**	**Azacitidine**	Epigenetic modulator	RICAZA (2016) Phase II trial, azacitidine single agent, n=37. Reduced GvHD (156). RELAZA (2012) Phase II trial, azacitadine single agent for mixed CD34+ chimerism, n=20. 80% responded (157). RELAZA2 (2018) Phase II trial, azacitadine single agent for MRD+ patients, n=55. Relapse free survival at 12 months 46% (84). Oran et al. (2020) Phase III trial, n=187. No difference in relapse free survival or overall survival (158).
	**Oral azacitidine**	Epigenetic modulator	On-going phase III trial NCT04173533 (oral azacitidine *versus* placebo).
	**Panobinostat**	Epigenetic modulator	On-going phase II trial NCT04326764
	**Lenalidomide**	Immunomodulator	LENAMAINT (2012) Phase II trial, n=10. Stopped early due to high incidence of severe acute GVHD (159).
**Targeted agents**	**Sorafenib**	Broad-spectrum tyrosine kinase inhibitor	SORMAIN study (2020) Randomised phase II, n=83, FLT3-ITD. Improved relapse free survival at 2 years (85% *versus* 53%) (160). Xuan et al. (13) Randomised phase III, n=202, FLT3-ITD. Reduced relapse at 1 year (7% *versus* 24%) (13).
	**Midostaurin**	Broad-spectrum tyrosine kinase inhibitor	RADIUS study (2020) Phase II, n=60 (161).
	**Gilteritinib**	FLT-3 inhibitor	On-going phase III trial NCT02997202 (gilteritinib *versus* placebo).
	**Venetoclax**	BCL-2 inhibitor	Kent et al. (2020) (abstract) Phase II, n=23. 6 month leukemia free survival: 87% (162). On-going trials Venetoclax + azacitidine. NCT04161885 (phase III) and NCT04128501 (phase II).
	**Glasdegib**	Hedgehog inhibitor	Kent et al. (2020) Phase II, n=31, high risk patients. No apparent benefit (163).
	**Ivosidenib**	IDH-1 inhibitor	On-going phase I trial NCT03728335
	**Enasidenib**	IDH-2 inhibitor	On-going phase I trial NCT03564821
**Cellular therapy**	**Prophylactic donor lymphocyte infusion (DLI)**	Graft-versus-leukemia effect	Schmid et al. (2019) Retrospective matched-pair study of prophylactic DLI for high-risk disease. Overall survival benefit (69.8% vs. 40.2%) (164). On-going phase II trial NCT02856464

##### Non-Targeted Agents

Non-targeted agents which modulate the immune system and tumor microenvironment have the advantage that they are generalizable, are not dependent on specific mutations and may maintain efficacy across the patchwork of clonally heterogeneous disease which is rapidly changing in the post-transplant bone marrow (165, 166).

Azacitidine is an epigenetic modulator that has efficacy in AML both as sole therapy and in combination with other treatments. Post-transplant, in the RICAZA study, Azacitadine was shown to be well tolerated and may both reduce the risk of GVHD through regulatory T-cell expansion and augment the GVL through upregulation of cancer associated antigens on leukemia cells (139, 156, 167). Azacitadine has also been studied in the RELAZA (157) and RELAZA2 (84) studies whereby patients were with mixed CD34+ chimerism and MRD positivity respectively were offered single-agent Azacitadine. In RELAZA, 80% patients responded and Azacitadine delayed relapse. In RELAZA2, relapse free survival at 12 months was 46% in those who had MRD detected and received Azacitadine, suggesting a delaying of haematological relapse. Despite this, a phase 3 RCT of azacitadine versus observation did not show evidence of survival benefit when used as post-transplant maintenance for patients with high risk AML, although this study was limited by the short duration of time that patients remained on treatment (158). The oral formulation of Azacitidine (CC-486) and Panobinostat, another epigenetic modulator have also shown promise in early phase studies and are both the subject of on-going RCTs (NCT04173533 and NCT04326764 respectively) (168, 169). Lenalidomide, an immunomodulator, in combination with Azacitidine is also active in post-transplant relapse (170) but is associated with GVHD when used as monotherapy in the maintenance setting (159) thus indicating the importance of studying the effects of drugs in this specific treatment stage.

DLI can induce remission in patients with hematological relapse, eradicate MRD and promote reversion to full donor chimerism. Alternatively, prophylactic DLI can be delivered to patients at high risk of relapse regardless of detectable disease. A recent observational, matched-pair study found that prophylactic DLI in patients with high-risk AML increased OS at five years by 30% (164). The on-going prospective, 2-arm, phase II PRO-DLI randomized trial will add valuable further information in this area (171). There are also developing technology to manipulate DLI to improve efficacy and limit toxicity. These are reviewed elsewhere, and studies are on-going (172).

#### Targeted Agents and Future Areas of Development

Routine application of NGS for DNA mutations have allowed for the identification of dysregulated, druggable pathways in AML. Many are only applicable to a subset of patients, but may also offer the first rung on the ladder of personalized medicine. The major challenges include identification of suitable, druggable targets in the context of clonal heterogeneity (165), and proving clinical efficacy when patient subgroups are relatively small.

An ever-expanding list of targeted treatments directed against key pathways in AML have received Food and Drug Administration FDA approval in recent years. FLT3, as described above is a tyrosine kinase, mutations in which are known to be associated with poor outcomes. In patients with FLT3 mutations, the use of post-transplant sorafenib, a broad-spectrum tyrosine kinase inhibitor (including FLT3), was associated with improved survival compared with placebo (13), findings that were consistent with the phase II SORMAIN study (160). As discussed earlier, the use of another broad-spectrum FLT3 inhibitor, midostaurin along with induction chemotherapy improves outcomes in FLT3-mutated AML (50). In the post-transplant setting, evidence of benefit from midostaurin is limited to a randomized phase II study (RADIUS) which showed a reduction in relapse with midostaurin treatment post-transplant albeit compared with historical controls (173).

Despite some evidence of benefit, there remain concerns about the off-target toxicity and adverse events associated with the broad-spectrum tyrosine kinase inhibitors. The aforementioned SORMAIN study found that the patients most likely to benefit from sorafenib post-transplant were those in whom MRD was detectable (160). For treatments where there are concerns over toxicity, especially in patients with more comorbidities, it is clear that post-transplant disease monitoring can add vital information for assessment of the risk-benefit equation. Second generation drugs which are potent, more specific FLT3 inhibitors are now available and have efficacy as monotherapy in relapsed AML (37). Clinical evaluation of Gilteritinib for post-transplant maintenance is underway (174).

Other targets of small molecule inhibitors include the anti-apoptotic protein BCL2, the Hedgehog signaling pathway, and isocitrate dehydrogenase 1 and 2 (IDH1 & 2). Venetoclax is a selective BCL2 inhibitor which is currently licensed in combination with Azacitidine for the treatment of older patients who are not suitable for intensive treatment and was found to have a substantial survival benefit in this cohort when compared with Azacitidine monotherapy (89). In a small study in the post-transplant maintenance setting, Venetoclax was reported to be safe and well tolerated but further studies are required to demonstrate benefit (162). Venetoclax is also being assessed in combination with Azacitadine as maintenance therapy post-transplant (175, 176) but its application may be limited by concerns over myelosuppression.

Glasdegib is an inhibitor of the Hedgehog signaling pathway which has evidence of modest benefit in combination with low dose Cytarabine for patients unfit for intensive treatment (38). It has been recently evaluated in a small single arm study in unselected high-risk patients in the post-transplant maintenance setting. However, there was no clear evidence of benefit either measured by MRD elimination, change in chimerism status, or clinical outcomes. Additionally, treatment was complicated by adverse events requiring pausing or cessation of treatment (163). Further studies in patients who are most likely to benefit as identified by genetic pre-stratification are required.

IDH1 and 2 are proteins which mediate the conversion of isocitrate to alpha-ketoglutarate. Gain in function mutations result in DNA and histone hypermethylation and altered downstream gene expression contributing to oncogenesis. Ivosidenib and Enasidenib, IDH1 and IDH2 inhibitors respectively both have evidence of efficacy in single-arm studies in AML (177–179) and are currently being evaluated for post-transplant maintenance (180, 181).

In summary, there is emerging, encouraging evidence that post-transplant maintenance therapies can reduce the risk of relapse, modulate the risk of GVHD, and improve survival. However, their use must be balanced in order to weigh up the additional toxicity and financial burden against the magnitude of the clinical effect. Detailed molecular analysis of a patient’s disease and post-transplant disease monitoring will allow further stratification and potentially identify the patients who are most likely to benefit from treatment (summarized in **Figure 3**).

## Conclusion

The establishment of large transplant trial networks has improved the scientific rationale behind transplant practice at every stage of the treatment pathway. This has improved the identification of which patients who are most likely to benefit from an allo-SCT, and also provides a rigorous assessment of novel agents that may benefit patients. Finally, by embedding correlative translational science in these studies, this further improves our knowledge and understanding of the scientific basis of clinical practice. This is of direct benefit to patients, and subsequently provides a vital starting place for future studies.

## Author Contributions

All authors contributed to the writing of this review article. All authors contributed to the article and approved the submitted version.

## Funding

Research support and clinical trials funding from CRUK, Bloodwise and Cure Leukaemia acknowledged. Core funding to the Birmingham ECMC Centre program is gratefully acknowledged.

## Conflict of Interest

CC has received honoraria from Celgene, Daichi-Sankyo, Novartis and Pfizer as well as research funding from Celgene. JL has received travel funding from Novartis and Daichi-Sankyo, honoraria from Pfizer, Janssen and Amgen.

The remaining author declares that the research was conducted in the absence of any commercial or financial relationships that could be construed as a potential conflict of interest.

The reviewer CS has declared past co-authorships with one of the authors CC, to the handling editor, at the time of review.
